# The Role of Dopamine Signaling and Neuroinflammation in Age‐Related Cognitive Dysfunction

**DOI:** 10.1002/brb3.71395

**Published:** 2026-04-14

**Authors:** Junior Bowen, Katie Hanna, Andrew M. J. Young, Gisela Helfer, Samantha L. McLean

**Affiliations:** ^1^ School of Pharmacy, Optometry and Medical Sciences University of Bradford Bradford UK; ^2^ School of Psychology and Vision Sciences University of Leicester Leicester UK

**Keywords:** aging, brain‐derived neurotrophic factor (BDNF), dopamine metabolism, neuroinflammation, prefrontal cortex, synaptic integrity

## Abstract

**Purpose**: Age‐related cognitive decline is a growing public health concern, yet early molecular indicators remain poorly defined. Since brain changes often precede behavioral symptoms, identifying early markers of vulnerability is critical. Here, we investigated whether dopamine regulation and synaptic or inflammatory signaling might provide early indicators of cognitive decline, prior to behavioral impairment.

**Method and Finding**: Female hooded‐Lister rats at 6 (young) and 12 (age‐unimpaired) months of age were tested using the novel object recognition (NOR) task, with no observable cognitive deficits found in either group. Biochemical analyses revealed marked molecular differences in the prefrontal cortex (PFC) of aged‐unimpaired rats. Synaptic proteins BDNF, PSD‐95, and synaptophysin were significantly reduced, indicating synaptic destabilization. Concurrently, expression of COMT and NET, key regulators of dopamine catabolism and reuptake, was increased, suggesting reduced dopaminergic tone. Inflammatory signaling also shifted: *Nfkb* and *Socs3* were increased at the transcriptional level in the PFC, while *Il‐6* and *Cox2* remained stable. In contrast, the hippocampus showed relative resistance to these changes, with no significant alterations in most markers, although NF‐κB activation was detected at the mRNA level, indicating posttranscriptional regulation.

**Conclusion**: Our findings suggest that the PFC undergoes a latent vulnerability phase during midlife, marked by synaptic and dopaminergic dysregulation alongside low‐grade inflammation, despite preserved cognitive performance. The hippocampus appears more resilient at this stage. Together, these early molecular changes may indicate later cognitive decline and offer a critical window for preventive intervention. Targeting these early shifts in the aging brain could hold transformative potential for delaying cognitive impairment.

## Graphical abstract

We reveal early disruption in synaptic integrity (PSD‐95, synaptophysin, BDNF), increased dopamine clearance (*Comt*, *Net*), and inflammatory signaling (*Nfkb*, *Socs3*) in the PFC, while the hippocampus remains largely resistant. These molecular “warning signs” precede behavioral deficits, highlighting potential targets for early intervention to protect brain health.

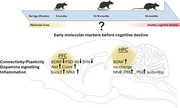

## 1 Introduction

Aging is associated with a range of accumulating changes that occur both peripherally and within the brain and central nervous system (CNS). As a result, aging increases susceptibility to several diseases, including cardiovascular disease, atherosclerosis, diabetes, and neurodegenerative conditions such as Alzheimer's, dementia, and Parkinson's disease (Gallizioli et al. 2023). Even in the absence of such diseases, healthy aging is often accompanied by cognitive decline, the severity of which varies between individuals and typically begins to emerge from middle age onward (Gallagher et al. 2011; Hughes et al. 2018; Wyss et al. 2000). This gradual decline is believed to result from age‐related structural and functional changes in the brain that often precede noticeable cognitive deficits (Beason‐Held et al. 2013; Resnick et al. 2003). Collectively, these accumulative changes are believed to underlie the deterioration of cognitive performance, commonly affecting memory, as well as processing speed and executive function (Fjell and Walhovd 2010).

Studies have shown that increasing age leads to neurodegenerative changes, resulting in reduced cognitive abilities, particularly affecting learning and memory (Nicholson et al. 2004). Episodic memory appears especially vulnerable due to dysfunction in the hippocampus, the surrounding perirhinal and entorhinal cortices, and the prefrontal cortex (PFC; Allen and Fortin 2013). Structural changes associated with aging have been specifically correlated with decline in cognitive, behavioral, and motor performances seen in elderly individuals. For example, dysregulated synaptic plasticity and reduced neurogenesis in the dentate gyrus of the hippocampus have been linked to deficits in episodic memory (Singhal et al. 2020). Similarly, reductions in gray matter and the loss of thin spines in layer 3 of the PFC (Burke and Barnes 2006) have been associated with a decline in executive function (Morrison and Baxter 2012; Nyberg 2017). Interestingly, the observed neurodegeneration changes in aging bear similarities to schizophrenia, which has been hypothesized as a potential syndrome of accelerated aging (Anthes 2014; Kirkpatrick et al. 2008; Schnack et al. 2016). This theory is supported by shared risk factors between the two conditions, such as cardiovascular abnormalities and increased mortality risk (Kirkpatrick et al. 2008; Nguyen et al. 2018).

An important component of episodic memory is recognition memory, which is defined by the ability to recognize previously encountered events, objects, or people (Myskiw et al. 2008). The novel object recognition (NOR) task is a commonly used behavior test in rodents to assess recognition memory. The neural substrates supporting object recognition memory depend on task parameters, particularly the length of the intertrial interval (ITI). Evidence from electrophysiological, lesion, and neuroimaging studies indicates that the perirhinal cortex plays a critical role in recognition memory following short ITIs, whereas hippocampal involvement becomes more pronounced at longer delays (>15 min) (Brown and Aggleton 2001; Clark et al. 2002; Hannesson et al. 2004). However, accumulating evidence also suggests a contribution of the PFC to recognition memory processes, including the encoding and evaluation of stimulus familiarity (Xiang and Brown 2004).

Dopamine is a critical neuromodulator involved in cognition, motivation, and reward processing, and age‐related declines in dopaminergic signaling are well‐documented, particularly within the PFC (Goldman‐Rakic and Brown 1981; Papenberg et al. 2025). The dopamine hypothesis of aging emerged from evidence showing reductions in dopamine receptor availability and changes in dopamine metabolism during healthy aging, which are closely linked to cognitive decline (Andersen et al. 2000; Naneix et al. 2012). Dopamine receptors are widespread and prominent within the CNS and are categorized into two families: the D1‐like family, which includes D1 and D5 receptors, and the D2‐like family, including D2, D3, and D4 receptors (Spano et al. 1978). Our previous work showed that dopamine D1 agonists improve cognition in a pharmacological model of cognitive dysfunction (McLean et al. 2009) and that young, healthy animals exhibit increased prefrontal dopamine levels during the retention trial of the NOR task (McLean et al. 2017), supporting optimal cognitive performance. However, with age, a decline in dopamine receptor density and binding potential has been observed across species, including humans and nonhuman primates, suggesting a reduction in extracellular dopamine availability in the aging PFC (Bäckman et al. 2000; Johansson et al. 2023; Papenberg et al. 2025). This is unknown in middle‐aged animals. Although dopamine synthesis capacity appears to remain relatively stable (Karrer et al. 2017), increased catechol‐O‐methyltransferase (COMT) activity with age may contribute to decreased dopamine levels (Tunbridge et al. 2007). Indeed, pharmacological inhibition of COMT has been shown to enhance cortical processing in both rodents and humans (Apud et al. 2006; Laatikainen et al. 2013; Tunbridge 2004), supporting its role in maintaining dopaminergic tone in the aging brain.

Interestingly, dopamine has been shown to have immunomodulating properties (Levite 2016), with dopamine receptors expressed on the surfaces of natural killer cells, microglia, and lymphocytes, all key mediators of the immune response (Sarkar et al. 2010). Dopamine can alter the expression of pro‐inflammatory cytokines such as tumor necrosis factor‐alpha (TNF‐α) and interleukin 6 (IL‐6) in a concentration‐dependent manner (Gaskill et al. 2012; Morimoto et al. 2022; Nolan et al. 2019). There is growing evidence supporting dopamine's role in neuroinflammation (A. Liu and Ding 2019; Ma and Ou 2023; Wang et al. 2018; Yan et al. 2015), with similar effects observed in peripheral tissues (Cao et al. 2020; J. Liu et al. 2021). While the age‐related decline of the dopaminergic system is well‐documented in both humans and animals, it remains unclear whether this natural decline coincides with coordinated changes in the central and peripheral immune systems. Furthermore, there is a notable lack of research into the timing of age‐related markers for synaptic integrity and dopaminergic signaling changes, as well as their overall predictive value for cognitive decline.

The correlation between dopaminergic signaling and inflammation raises critical questions about how these processes interact in the context of aging and cognitive decline. Chronic inflammation has been closely associated with age‐related diseases, including cardiovascular disease and type 2 diabetes (Libby 2002; Soysal et al. 2016; Tsalamandris et al. 2019), and more recently, with cognitive decline (Leonardo and Fregni 2023). Pro‐inflammatory markers such as interleukin 1β (IL1β), IL‐6, and TNF‐α are consistently upregulated in healthy elderly individuals (Fuchs et al. 2013; Hager et al. 1994; Karim et al. 2014; Leonardo and Fregni 2023; McAfoose et al. 2009; Roubenoff et al. 1998; Wei et al. 1992), a phenomenon termed “inflammaging” (Franceschi and Campisi 2014). This chronic inflammatory state has been associated with reduced cognitive performance, particularly in tasks involving executive function (Bradburn et al. 2017; Kálmán et al. 2009; Kim et al. 2017; Mooijaart et al. 2013; Singh and Newman 2011; Tegeler et al. 2016; Trollor et al. 2012; Weaver et al. 2002) and memory‐based tasks (Serre‐Miranda et al. 2020). Animal models with induced chronic inflammatory signaling provide robust evidence that this state not only accelerates the aging process but also exacerbates age‐related pathologies and cognitive impairments (Neves and Sousa‐Victor 2019; Jurk et al. 2014; Bernal et al. 2014; Franceschi et al. 2017; Buchanan et al. 2008), specifically memory (Czerniawski et al. 2015; Hovens et al. 2014; Laurent et al. 2017; Michels et al. 2015; Reis et al. 2017; Wang et al. 2018). How chronic inflammation may affect cognition has been explored; one possible mechanism by which this may occur is through pro‐inflammatory cytokines, which have been shown to affect neurotrophins such as brain‐derived neurotrophic factor/nerve growth factor (BDNF/NGF), which have a known role in the consolidation of memory and have been shown to decrease with aging within the hippocampal CA1 and CA3 regions (Chapman et al. 2012; Cortese et al. 2011; Richwine et al. 2008). Inflammation also reduces BDNF in the PFC after poly I:C treatment, which induces chronic inflammation when given to young rats (Gibney et al. 2013; Guan and Fang 2006) and may contribute to the reduction in neurogenesis seen from middle age onward in rats and mice (Solano Fonseca et al. 2016).

While it is known that changes in the brain precede the onset of cognitive deficits in humans (Beason‐Held et al. 2013; Resnick et al. 2003), the specific molecular indicators of early cognitive decline remain unclear. To address this, we investigated potential early molecular changes preceding observable behavioral modifications in an animal model. We hypothesize that changes in dopamine receptor expression and dopamine metabolism, as well as inflammatory markers in the PFC or hippocampus, occur before behavioral changes are observed. The expression of synaptic markers, such as synaptophysin, postsynaptic density 95 (PSD‐95), and BDNF, may be dependent on these early molecular changes. To test this, we examined cognition using the NOR task in young (6 months) and age‐unimpaired (AUI) (12 months) female hooded‐Lister rats, followed by postmortem biochemical analysis of tissues from the PFC and hippocampus.

## 2 Methods

### 2.1 Animals and Ethics

Twenty female Lister Hooded rats were obtained from Charles River (Kent, UK) at 5–6 weeks of age (supplier‐reported weight range: 120–140 g) and were maintained under standard housing conditions in the animal facility at the University of Bradford. Behavioral testing was performed when the rats were either 6 months old (referred to as the young group) or 12 months old (referred to as the AUI group). Rats were housed in groups of four in Type III rat cages (Arrowmight, Hereford, UK) enriched with soft woodchip bedding (Datesand, Manchester, UK), shredded paper (Sizzle Nest, Datesand, Manchester, UK), and a red plastic tunnel for enrichment. Housing conditions were maintained at a temperature of 21°C ± 5°C, humidity of 50 ± 5%, and a 12:12‐h light/dark cycle, with an average light intensity of 150 lux. Filtered tap water and a standard pelleted chow diet (Teklad 2018 Global 18% Protein Rodent Diet Envigo, Loughborough, UK) were provided ad libitum. All animal experiments were performed in accordance with the UK Animals (Scientific Procedures) Act 1986 and approved by the Animal Welfare Ethical Review Body at the University of Bradford (Home Office Project License number: P6D8A22DC, UK).

### 2.2 Behavioral Testing

NOR was carried out in female young and AUI rats (*n* = 10/group). All behavioral testing was carried out during the first half of the light cycle. The arena was located in the experimental room under standard ambient lighting conditions, with no additional illumination applied. Care was taken to position the arena such that no shadows were cast within the testing area. We used female rats in this study as evidence from our laboratory suggests that females can outperform male rats in NOR (Sutcliffe et al. 2007) and that the stage of the estrous cycle does not affect cognitive performance in NOR or other cognitive tests performed in our laboratory in drug‐naïve rats (McLean et al. 2009; Sutcliffe et al. 2007). The number of animals per group was determined using power calculations based on results from our previous behavioral studies (McLean et al. 2016; McLean et al. 2021; Yun et al. 2022). NOR was conducted separately for each individual rat, and the location of the novel object was randomly assigned. Rats were habituated to the NOR arena (52‐cm wide × 40‐cm high × 52‐cm long) for 20 min for three consecutive days prior to testing day. Testing day consisted of a 3‐min habituation session, with the rat placed in the NOR arena, followed by an acquisition trial in which two identical objects were placed in opposite corners, a minimum of 6 cm from the side wall of the NOR box, with the animal for a period of 3 min. The rat was then removed and placed back in the home cage with cage mates for a 1‐min ITI. The arena was cleaned with 70% ethanol, and the rat was reintroduced to the arena for the retention trial for 3 min with a novel object and an unused triplicate version of the familiar object (previously seen in the acquisition trial).

All experiments were video recorded for subsequent analysis of behavior. The exploration time (s) of each object in each phase was recorded manually using two stopwatches, and the discrimination index was calculated: discrimination index (DI) = (time exploring the novel object [s] − time exploring the familiar object) / total time exploring both novel and familiar objects. The DI represents the difference in exploration time expressed as a proportion of the total time spent exploring the two objects in the retention trial. Locomotor activity was quantified by scoring the total number of lines crossed by the rat during both acquisition and retention trials.

### 2.3 Protein Analysis

Rats were euthanized and brains were removed 1–4 days following behavioral testing. Dissected PFC and hippocampal regions were homogenized in chilled RIPA Lysis Buffer (Millipore, MA, USA) supplemented with PhosSTOP and cOmplete Mini, EDTA‐Free Protease Inhibitor Cocktail (Merck, Darmstadt, Germany), according to the instructions, and 0.5 M sucrose solution, using a Potter–Elvehjem homogenizer, with the right hippocampal hemisphere used for protein analysis (i.e., subregions were not separated). Homogenates were centrifuged at 1000 × *g* for 10 min at 4°C, followed by re‐centrifugation of the supernatant at 15,000 × *g* for 30 min at 4°C. Protein concentration was determined using the Bradford assay (Sigma Aldrich, MO, USA), and absorbance was measured on a FlexStation3 microplate reader (Molecular Devices).

Equal amounts of protein (30–150 µg/lane) were mixed with 4× loading buffer (Novex, CA, USA) and boiled at 95°C for 5 min. SDS‐PAGE was performed at 100 V on 12%–12.5% polyacrylamide gels, and proteins were transferred to nitrocellulose membranes (0.45 µm; Amersham Pharmacia Biotech) using the wet transfer method (Bio‐Rad, CA, USA). Membranes were blocked in 5% nonfat dry milk in TBS‐T (10 mM Tris‐HCl [pH 7.2], 250 mM NaCl, 0.05% Tween‐20) and incubated with a primary antibodies: synaptophysin (1:10,000; Synaptic Systems, Gottingen, Germany), PSD95 (1:10,000; Sigma–Aldrich, Gottingen, Germany), and BDNF (1:10,000; Aviva Systems Biology, CA, USA) overnight at 4°C. Membranes were then washed three times in TBS‐T before incubation for 1 h with the appropriate secondary antibody. GAPDH (1:20,000; Proteintech, IL, USA) was used as a loading control for PSD95 and BDNF, whereas α‐Tubulin (1:10,000; DSHBY, IA, USA) was used as a loading control for synaptophysin, as synaptophysin has a molecular weight similar to GAPDH and could interfere with band resolution and quantification if probed on the same membrane. These housekeeping proteins were used for within‐blot normalization to control for loading variability. Membranes were washed again three times in TBS‐T before administering clarity ECL (Biorad, CA, USA). Band intensity was measured using the Bio‐Rad ChemiDoc imaging system. BDNF, PSD‐95, and synaptophysin were each analyzed on separate gels, with the appropriate loading control run on the same gel and membrane. Each band represents a protein derived from a single individual animal (young or AUI). PSD‐95 was detected as a doublet, and both bands were included in the quantification. Band intensities were additionally normalized to a pooled homogenate of young cortical tissue run on each membrane to allow comparison across gels, providing a consistent between‐blot reference. Only young tissue was included in this pooled control to avoid potential age‐related variability in housekeeping protein expression.

### 2.4 RNA Preparation and Expression

For each animal, the right hemisphere was consistently used for protein analysis, while the left hemisphere was used for RNA extraction. This assignment was not counterbalanced. Within each assay, the same hemisphere was used for both the PFC and hippocampus to maintain consistency across tissues. Samples were homogenized in cell disruption lysis buffer (Millipore, MA, USA) using 1.5‐mm zirconium beads (Triple‐Pure) and a BeadBug microtube homogenizer. The supernatant was extracted, and RNA isolation was performed according to the manufacturer's protocol for the Aurum Total RNA Mini Kit (Bio‐Rad, CA, USA). RNA concentration was quantified using a Nanodrop Lite spectrophotometer (Thermo Fisher Scientific, UK). Subsequently, 500 ng of purified RNA was reverse transcribed into cDNA using the Applied Biosystems cDNA Synthesis Kit (Thermofisher, Lithuania).

Gene expression levels were quantified using qPCR with iTaq Universal SYBR Green Supermix (Bio‐Rad, CA, USA) on a T100 thermal cycler (Bio‐Rad, CA, USA). The amplification program consisted of 40 cycles of 2 min at 50°C, 5 min at 95°C, 10 s at 95°C, and 30 s at 60°C. Relative gene expression levels were normalized to D‐Box gene expression, and fold changes were calculated using the 2^−ΔΔ^ *^Ct^* method. Primer sequences (Sigma–Aldrich, Darmstadt, Germany) were as follows: *D1*—forward: *CTTCTGGAAGATGGCTCCTAAC*, reverse: *CAGAGTCCATGCTACGCTAATC*; *D2*—forward: *CAACAATACAGACCAGAATCAG*, reverse: *GGAGGACGATGTAGATTTTG*; *COMT*—forward: *TACAGGACAAAGTCACCATC*, reverse: *CAAGAAAGACCATGTCTAGTG*; *MOAB*—forward: *GTCAAGTGAGTGAGCGGATAAA*, reverse: *CTCCAGGAAGGTGTTGGTAATG*; *NET*—forward: *TAAGAAGTCAGGTCCAGCACC*, reverse: *AGTAGAGCAAGGAAGGCACC*; *COX‐2*—forward: *TCTCCAACCTCTCCTACTACAC*, reverse: *CTCCACCGATGACCTGATATTT*; *SOCS3*—forward: *TTCTTTACCACCGACGGAAC*, reverse: *CAGCTGGGTCACTTTCTCATAG*; *Il‐6*—forward: *CCGTTTCTACCTGGAGTTTGT*, reverse: *GTTTGCCGAGTAGACCTCATAG*.

### 2.5 ELISA

Whole‐cell extracts were prepared according to the TransAM kit (TransAM, CA, USA) instructions and quantified using the Bradford assay (Sigma–Aldrich, CA, USA). Briefly, a total of 5 µg per well of each sample was added to a 96‐well assay plate coated with an oligonucleotide containing the NF‐κB consensus‐binding site (5′‐GGGACTTTCC‐3′). Primary antibodies against p65 and p50 were then applied, followed by HRP‐conjugated secondary antibodies. Absorbance was measured using the FlexStation 3. Results were normalized to the kit‐provided control (*Raji* nuclear extract).

### 2.6 Statistics

All figures were created and statistical analysis was performed using GraphPad Prism version 9 (GraphPad Software, USA). All statistical tests were two‐tailed, and differences were considered statistically significant at *p* < 0.05. Normality was assessed using the Shapiro–Wilk test, and all data met the assumptions of normal distribution. Group comparisons were performed using unpaired *t*‐tests. For each comparison, equality of variances was assessed using an *F*‐test. Where variances were significantly different, Welch's correction was applied. In all cases, the outcome of statistical significance (significant or nonsignificant) remained the same. Data are presented as mean ± SD.

## 3 Results

### 3.1 Preserved Recognition Memory in Young and AUI Rats

**FIGURE 1:**
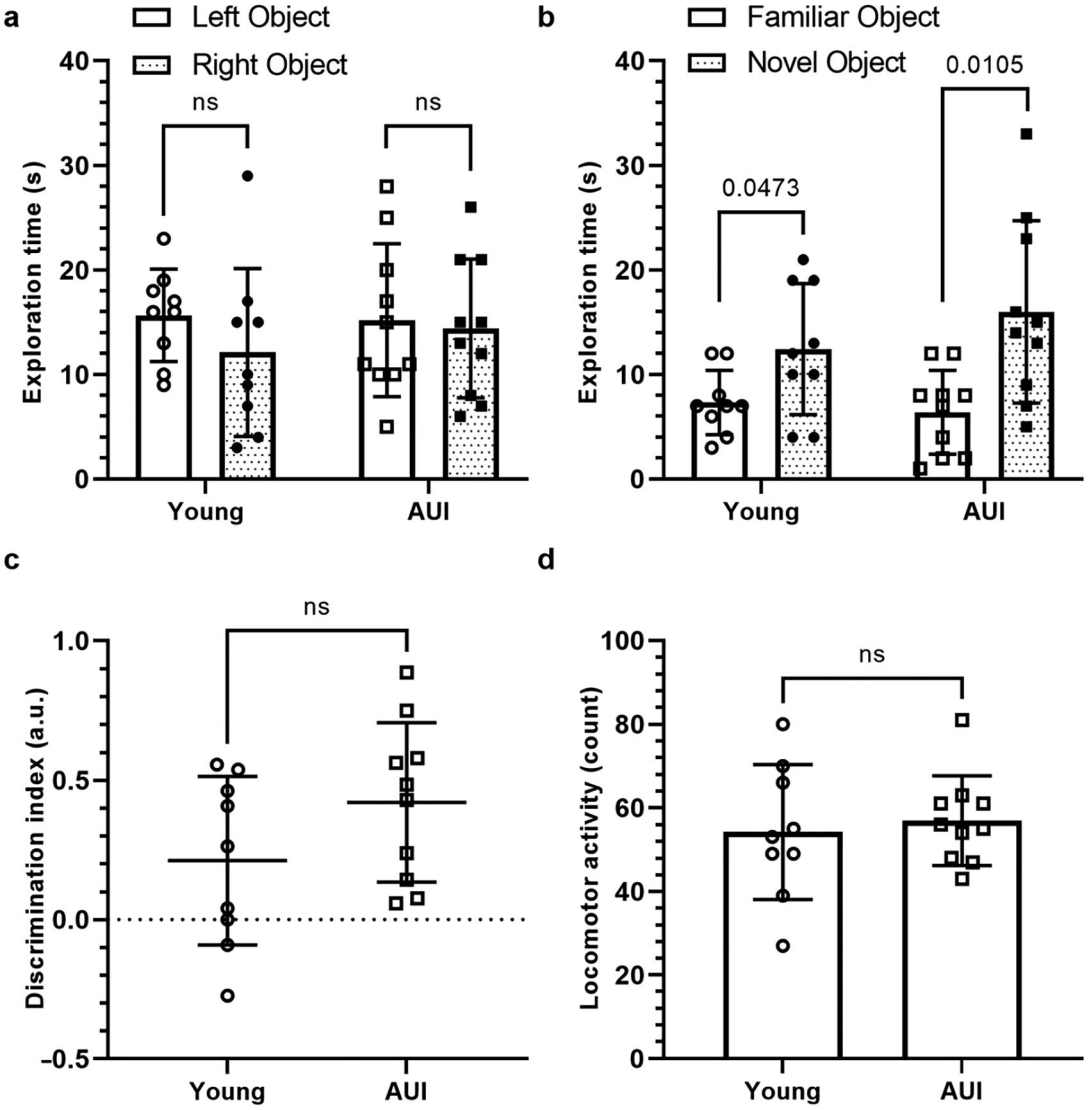
Novel object recognition (NOR) performance in young and age‐unimpaired (AUI) rats. (a) Both groups explored the left and right objects equally during the acquisition phase (young: *t*(8) = 1.380, *p* = 0.2050; AUI: *t*(9) = 0.4143, *p* = 0.6884). (b) Both groups showed a preference for the novel object during the retention phase (young: *t*(8) = 2.342, *p* = 0.0473; AUI: *t*(9) = 3.219, *p* = 0.0105). (c) Discrimination index did not differ significantly between groups (*t*(17) = 1.553, *p* = 0.1389). (d) Locomotor activity (total line crossings) was similar between groups (*t*(17) = 0.4304, *p* = 0.6723). Each point represents an individual animal, and bars indicate the group mean ± SD. One young rat was excluded for not exploring either object during acquisition (young: *n* = 9, AUI: *n* = 10). Statistical analysis was performed using two‐tailed paired *t*‐tests (a and b) or unpaired *t*‐tests (c and d), and data were normally distributed.

To confirm that there was no difference in recognition memory, short‐term memory was measured using the NOR in both young (*n* = 9) and AUI (*n* = 10) groups. One animal from the young group was excluded as it failed to explore either object (left or right) during the acquisition stage. Both groups explored both objects equally within the acquisition stage with no significant preference for object position (left vs. right) (Figure 1a; young: left vs. right *t*(8) = 1.380, *p* = 0.2050; AUI: left vs. right *t*(9) = 0.4143, *p* = 0.6884). Within the retention stage, both groups exhibited a preference for the novel object compared to the familiar object, as indicated by significantly longer exploration time of the novel object (Figure 1b; young: novel vs. familiar *t*(8) = 2.342, *p* = 0.0473; AUI: novel vs. familiar *t*(9) = 3.219, *p* = 0.0105). Although there is a slight increase in the discrimination index of the AUI group compared to the young group, this did not approach statistical significance (Figure 1c; *t*(17) = 1.553, *p* = 0.1389). Throughout the trials, locomotor activity was recorded to assess age‐related effects on rats’ locomotor ability to explore objects. Data show no significant difference between the groups, indicating that age did not affect locomotor activity (Figure 1d; *t*(17) = 0.4304, *p* = 0.6723). There were no observed significant differences in total object exploration time in acquisition or retention stages of the test (data not shown). Both young and AUI rats showed no observable impairment in NOR, demonstrated by a lack of preference for the novel object, and therefore recognition memory is still intact at 12 months.

### 3.2 Age‐Related Changes in Synaptic Protein Expression in the PFC and Hippocampus

**FIGURE 2:**
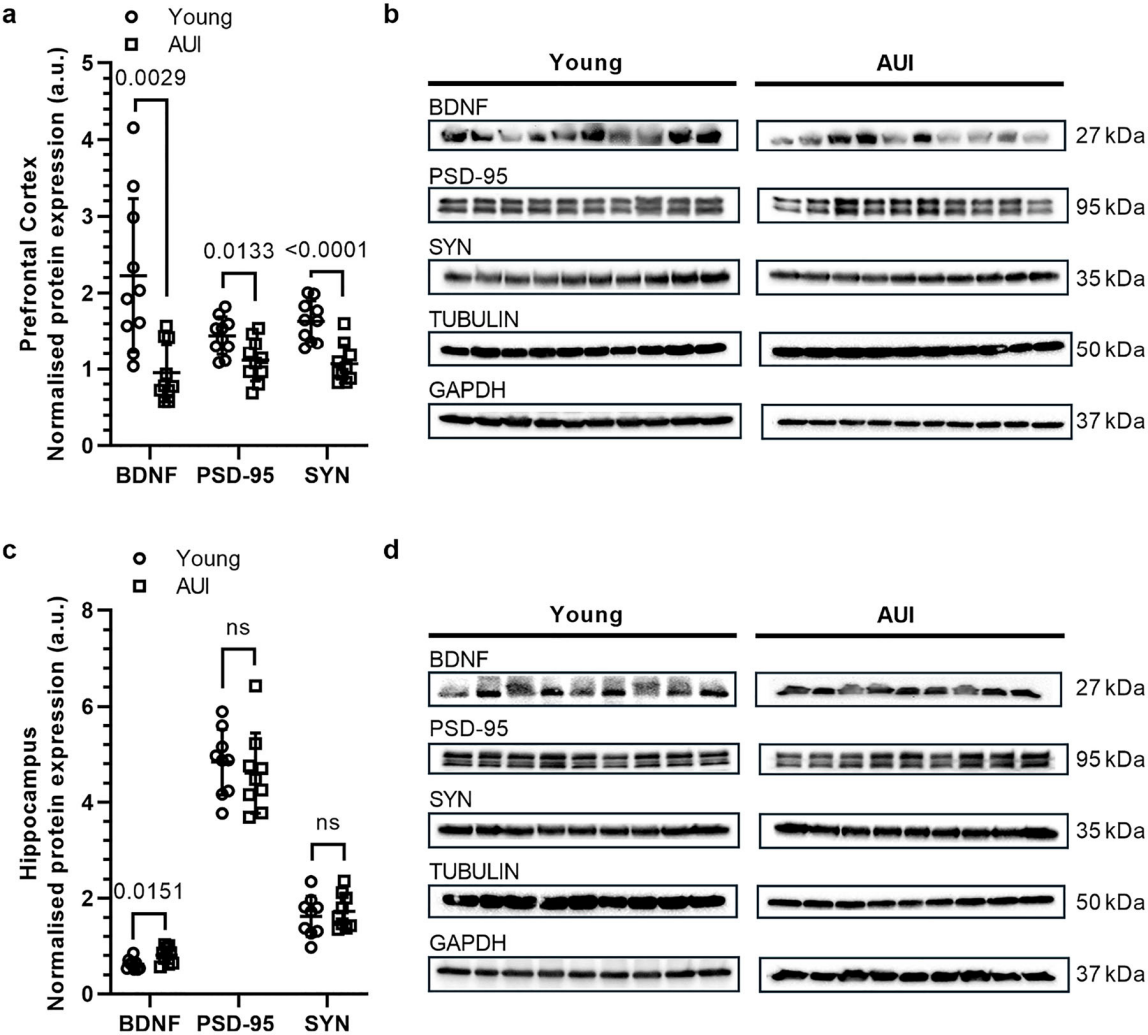
Age‐related changes in synaptic protein expression in the prefrontal cortex (PFC) and hippocampus. (a) Quantification of synaptic protein levels in PFC lysates shows that pro brain‐derived neurotrophic factor (proBDNF; *t*(11.52) = 3.759, *p* = 0.0029), postsynaptic density protein 95 (PSD‐95; *t*(18) = 2.746, *p* = 0.0133), and synaptophysin (SYN; *t*(18) = 4.972, *p* < 0.0001) are significantly downregulated in AUI rats compared to control. (b) Representative western blot images for PFC proteins. (c) In hippocampal lysates, proBDNF is significantly upregulated (*t*(16) = 2.722, *p* = 0.00151), whereas PSD‐95 (*t*(16) = 0.6393, *p* = 0.5317) and SYN (*t*(16) = 0.5481, *p* = 0.5912) do not change. (d) Representative western blot images for hippocampal proteins. Protein levels were normalized to α‐Tubulin or GAPDH (see Section 2). Each point represents an individual animal, and horizontal lines indicate the group mean ± SD, *n* = 9/group. Statistical analysis was performed using a two‐tailed unpaired *t*‐test, and data were normally distributed.

Next, we investigated molecular markers of synaptic density in the PFC and hippocampus to establish protein changes directly related to presynaptic (synaptophysin) and postsynaptic (PSD‐95) integrity and to assess the overall health of synaptic connections. Western blot analysis was performed on established indicators of synaptic density in these brain regions. In the PFC, BDNF, PSD‐95, and synaptophysin levels were significantly decreased in AUI rats compared to young rats (Figure 2a; BDNF: *t*(11.52) = 3.759, *p* = 0.0029; PSD‐95: *t*(18) = 2.746, *p* = 0.0133; SYN: *t*(18) = 4.872, *p* < 0.001). In contrast, the hippocampus showed a different pattern, with BDNF levels significantly increased in AUI rats compared to young rats (Figure 2c; *t*(16) = 2.722, *p* = 0.0151), but PSD‐95 (*t*(16) = 0.6393, *p* = 0.5317) and synaptophysin levels (*t*(16) = 0.5481, *p* = 0.5912) showed no significant age‐related differences.

### 3.3 Molecular Markers of Dopaminergic Function in Aging Brain Regions

To examine potential age‐related changes in the dopaminergic system, we quantified mRNA expression of key dopamine pathway genes in the PFC and hippocampus using qPCR. This molecular approach was chosen due to the limited specificity and reliability of available antibodies for dopamine receptors D1 and D2 (Bodei et al. 2009; Stojanovic et al. 2017). In the PFC, expression of *Drd1*, *Drd2*, *Comt*, *Net*, and *Mao‐B* was measured, while in the hippocampus, *Drd1*, *Drd2*, *Comt*, *Net*, and *DAT* were examined. Gene selection was informed by region‐specific expression patterns: DAT has been shown to be sparsely expressed in the PFC (Sesack et al. 1998), while DAT and NET are the primary dopamine clearance mechanisms in the hippocampus (Borgkvist et al. 2012).

**FIGURE 3:**
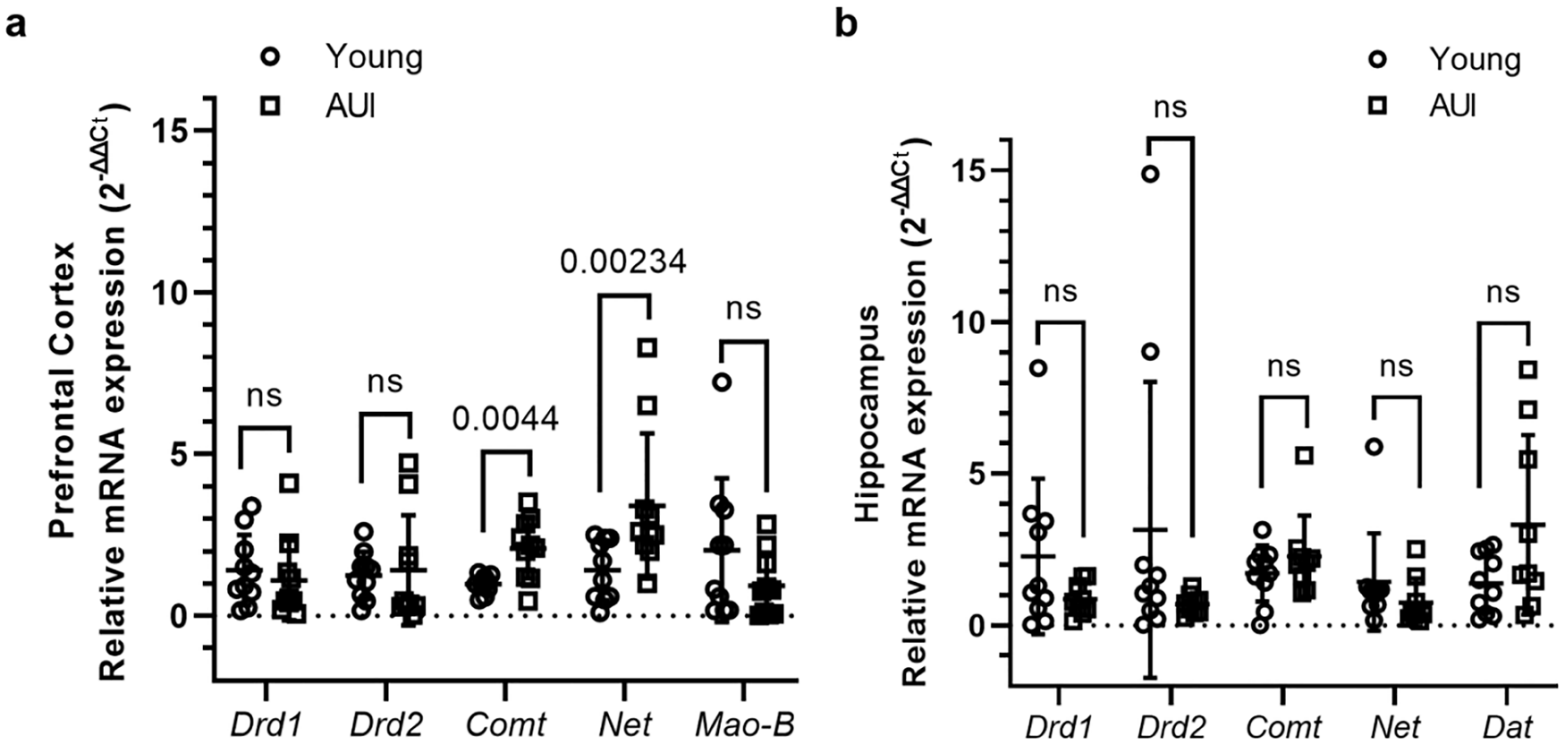
Age‐related changes in dopaminergic and catecholaminergic gene expression in the prefrontal cortex (PFC) and hippocampus. (a) Relative mRNA expression of *Drd1*, *Drd2*, *Comt*, *Net*, and *Mao‐B* in the PFC of young and age‐unimpaired (AUI) rats. *Drd1* (*t*(18) = 0.6066, *p* = 0.5517), *Drd2* (*t*(12.19) = 0.2663, *p* = 0.7945), and *Mao‐B* (*t*(12.4) = 1.428, *p* = 0.1779) showed no significant difference between groups, while *Comt* (*t*(10.54) = 3.610, *p* = 0.0044) and *Net* (*t*(12.02) = 2.594, *p* = 0.0234) were significantly increased in AUI rats compared to young control. (b) Relative mRNA expression of *Drd1* (*t*(9.865) = 1.686, *p* = 0.1231), *Drd2* (*t*(9.089) = 1.588, *p* = 0.1465), *Comt* (*t*(17) = 1.072, *p* = 0.2989), *Net* (*t*(17) = 1.141, *p* = 0.2697), and *Dat* (*t*(9.552) = 1.866, *p* = 0.00930) showed no significant difference between groups in the hippocampus. Expression levels were normalized to *D‐box* and calculated using the 2^−ΔΔ^ *^Ct^* method. Each point represents an individual animal, and horizontal lines indicate group mean ± SD. Statistical analysis was performed using two‐tailed unpaired *t*‐tests, *n* = 9–10/group. One animal was excluded due to incomplete tissue recovery. *Comt*, catechol‐O‐methyltransferase; *Net*, norepinephrine transporter; *Drd1*, dopamine receptor D1; *Drd2*, dopamine receptor D2; *Mao‐B*, monoamine oxidase B; *Dat*, dopamine transporter.

In the PFC, mRNA levels of *Comt* (*t*(10.54) = 3.610, *p* = 0.0044) and *Net* (*t*(12.02) = 2.594, *p* = 0.0234) were significantly increased in AUI rats compared to young controls, with no significant differences observed in *Drd1* (*t*(18) = 0.6066, *p* = 0.5517), *Drd2* (*t*(12.19 = 0.2663, *p* = 0.7945), or *Mao‐B* (*t*(12.4) = 1.428, *p* = 0.1779) expression (Figure 3a). In the hippocampus, expression levels of *Drd1* (*t*(9.865) = 1.686, *p* = 0.1231), *Drd2* (*t*(9.089) = 1.588, *p* = 0.1465), *Comt* (*t*(17) = 1.072, *p* = 0.2989), *Net* (*t*(17) = 1.141, *p* = 0.2697), and *Dat* (*t*(9.552) = 1.866, *p* = 0.0930) did not differ significantly between age groups (Figure 3b).

### 3.4 Region‐Specific Inflammatory Signaling and NF‐kB Activation

**FIGURE 4:**
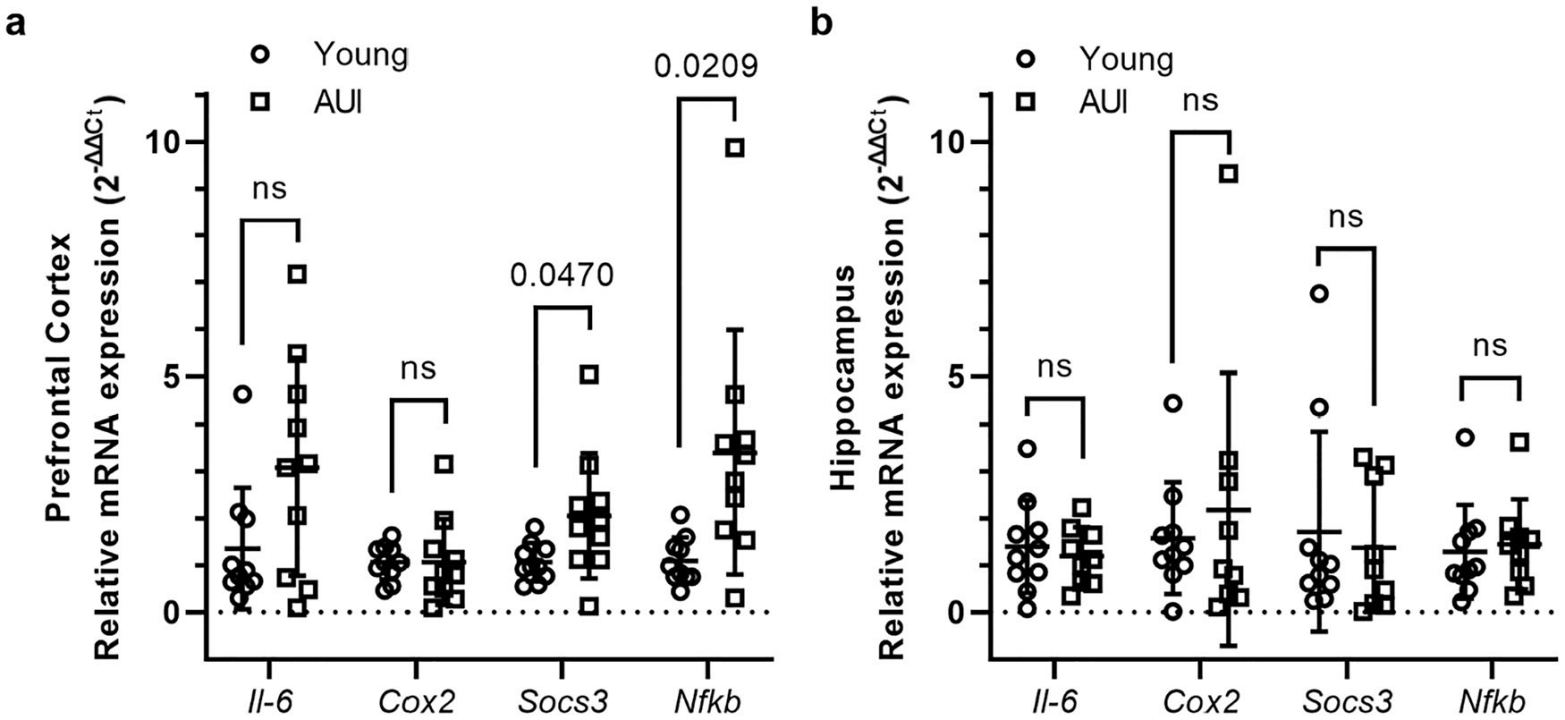
Age‐related changes in inflammatory gene expression in the prefrontal cortex (PFC) and hippocampus. (a) Relative mRNA expression of *Il‐6*, *Cox2*, *Socs3*, and *Nfkb* in the PFC of young and age‐unimpaired (AUI) rats. *Il‐6* (*t*(18) = 2.065, *p* = 0.0536) and *Cox2* (*t*(11.90) = 0.006415, *p* = 0.9950) showed no significant difference between groups, while *Socs3* (*t*(10.65) = 2.246, *p* = 0.0470) and *Nfkb* (*t*(9.660) = 2.757, *p* = 0.0209) were significantly increased in AUI rats compared to young controls. (b) Relative mRNA expression of *Il‐6*, *Cox2*, *Socs3*, and *Nfkb* in the hippocampus. No significant differences were observed between young and AUI rats for any gene (*Il‐6*: *t*(17) = 0.5266, *p* = 0.6053; *Cox2*: *t*(10.39) = 0.5795, *p* = 0.5746; *Socs3*: *t*(17) = 0.4139, *p* = 0.6841; *Nfkb*: *t*(17) = 0.3590, *p* = 0.7240). Expression levels were normalized to *D‐box* and calculated using the 2^−ΔΔ^ *^Ct^* method. Each point represents an individual animal, and horizontal lines indicate group mean ± SD. Statistical analysis was performed using two‐tailed unpaired *t*‐tests, *n* = 9–10/group. One animal was excluded due to incomplete tissue recovery. *Il‐6*, interleukin‐6; *Cox2*, cyclooxygenase‐2; *Socs3*, suppressor of cytokine signaling 3; *Nfkb*, nuclear factor kappa B.

Given the well‐established association between inflammation and aging, we next examined mRNA expression of key pro‐ and anti‐inflammatory markers (*Il‐6*, *Nfkb*, *Cox2*, *and Socs3*) in both the PFC and hippocampus. In the PFC, we found a significant upregulation of *Nfkb* (*t*(18) = 2.757, *p* = 0.0130) and *Socs3* (*t*(10.65) = 2.246, *p* = 0.0470) in AUI rats compared to young controls, suggesting enhanced inflammatory signaling and potential dysregulation of negative feedback mechanisms. No significant differences were detected in *Il6* (*t*(18) = 2.065, *p* = 0.0536) or *Cox2* (*t*(11.90) = 0.006415, *p* = 0.9950) expression in this region (Figure 4a). In contrast, none of the inflammatory markers showed differential expression in the hippocampus (*Il‐6*: *t*(17) = 0.5266, *p* = 0.6053; *Cox2*: *t*(10.39) = 0.5795, *p* = 0.5746; *Socs3*: *t*(17) = 0.4139, *p* = 0.6841; *Nfkb*: *t*(17) = 0.3590, *p* = 0.7240; Figure 4b).

**FIGURE 5:**
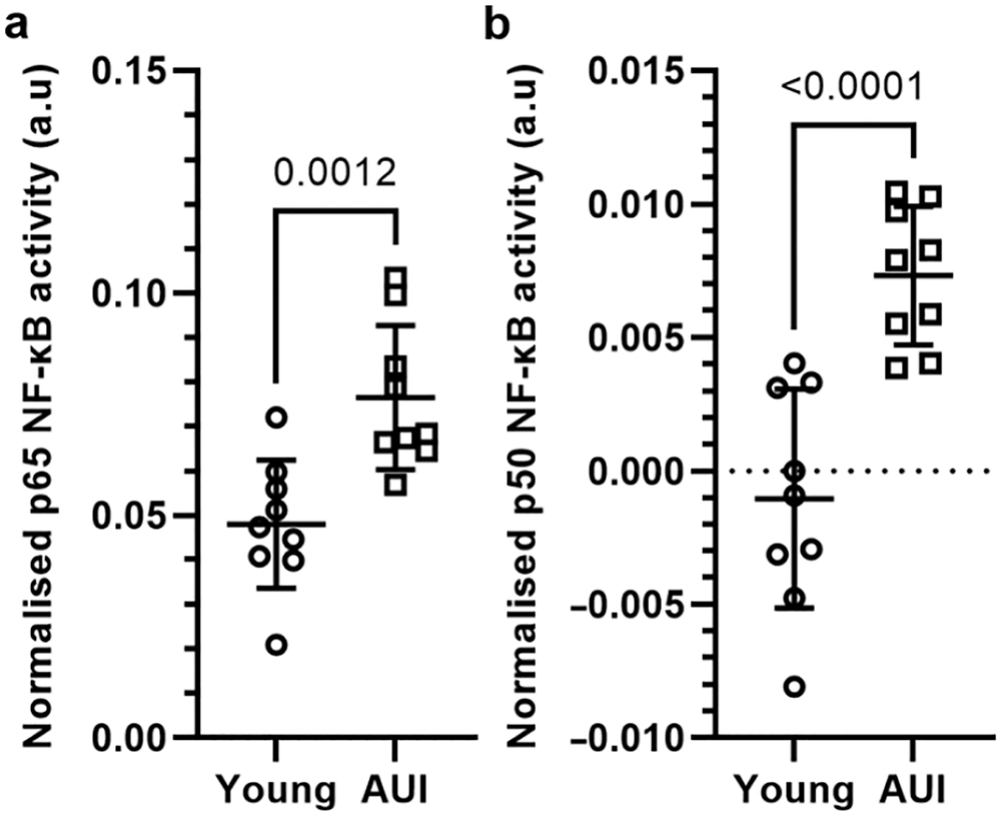
Increased NF‐κB DNA‐binding activity of P65 and P50 subunits in the hippocampus of age‐unimpaired (AUI) rats. Activated levels of NF‐kB were quantified in hippocampal extracts from young and AUI rats using a DNA‐binding assay. Each point represents an individual animal, and horizontal lines indicate the group mean ± SD, *n* = 9/group. Statistical analysis was performed using a two‐tailed unpaired *t*‐test, and data were normally distributed. (a) P65 was significantly higher in AUI rats compared to young controls (*t*(16) = 3.943, *p* = 0.0012). (b) P50 activity was also significantly higher in AUI rats compared to young controls (*t*(16) = 5.172, *p* < 0.0001).

Given the increased *Nfkb* mRNA expression in the PFC and the absence of corresponding transcriptional changes in the hippocampus, we next examined whether age‐related regulation of NF‐κB in the hippocampus might occur at the level of transcription factor activation rather than gene expression. To address this, we performed a DNA‐binding assay to quantify NF‐κB signaling in hippocampal tissue, measuring nuclear levels of the p56 and p50 subunits. Interestingly, NF‐κB‐binding activity was significantly increased in the AUI hippocampus compared to young controls (p65: *t*(16) = 3.943, *p* = 0.0012 and p50: *t*(16) = 5.172, *p* < 0.0001), indicating increased activation of this pathway despite stable mRNA expression levels (Figure 5).

## 4 Discussion

While it is known that changes in the brain precede the onset of cognitive deficits in humans (Beason‐Held et al. 2013; Resnick et al. 2003), the specific molecular indicators of early cognitive decline remain unclear. To address this, we sought to uncover early molecular indicators of cognitive decline that precede observable behavioral changes. As hypothesized, we identified region‐specific alterations in gene and protein expression in the PFC and hippocampus of AUI rats, despite the absence of behavioral differences in the NOR task, indicating that their cognitive performance remained intact, as also observed in young animals. We found changes in dopamine metabolism, inflammatory markers, and synaptic proteins. Importantly, the presence of these molecular changes in the absence of detectable behavioral deficits should not be interpreted as evidence of dysfunction. Rather, they reflect age‐associated molecular alterations that may represent early divergence or vulnerability within specific brain regions, occurring while cognitive performance remains preserved. These results highlight the complexity of age‐related brain changes and suggest that molecular alterations may serve as early indicators of future cognitive decline.

To identify early molecular indicators, it was important to select rats at ages comparable to young (18–35 years) and middle‐aged (36–55 years) humans with intact cognitive function (Sengupta 2013). Our choice of 6‐month‐old (young) and 12‐month‐old (AUI) rats proved appropriate, as these rats showed no impairment in cognition or reduction in locomotor activity. The selection of these age groups is well‐supported by previous research. Typically, young rats (3–6 months) show no differences in various behavioral tests, including NOR and spatial object recognition (Antunes and Biala 2012; Forbes et al. 2014). Rats are generally considered aged at 18–24 months, equivalent to >56 years in humans (Sengupta 2013). Our 12‐month‐old group represents a midpoint between young and aged rats, corresponding to middle‐aged humans. This age selection aligns with established correlations between rat and human ages, where in adulthood, one rat month is approximately equivalent to 2.5–3 human years (Sengupta 2013). Our behavioral data confirmed that recognition memory remains intact in 12‐month‐old rats, as no cognitive deficits were detected. Thus, we refer to these rats as AUI, highlighting the absence of overt cognitive impairment at this age. This observation supports the notion that working memory and executive control functions tend to decline earlier than recognition memory during aging (Erixon‐Lindroth et al. 2005). It is important to note that the trajectory of cognitive aging can be influenced by modifiable lifestyle risk factors and neurodegenerative processes. For example, we have previously shown that rats exposed to a high‐fat diet (Yun et al. 2022) or intracerebroventricular administration of soluble amyloid β oligomers (Watremez et al. 2018) show impairments in NOR at a much younger age. In the present study, by selecting rats not exposed to such risk factors or models of neurodegeneration, we ensured that the molecular changes observed reflect normal aging processes rather than pathological conditions.

To investigate molecular correlates of age‐related cognitive decline, we examined proteins associated with synaptic structure and function in the PFC. Our analysis revealed a synaptic decline in the PFC of AUI rats, with significant downregulation of BDNF (a neurotrophin), PSD‐95 (a postsynaptic scaffolding protein), and synaptophysin (a presynaptic vesicle protein). The decline in BDNF is especially relevant, as BDNF is crucial for maintaining synaptic strength, promoting dendritic growth, and supporting plasticity via activation of the TrkB receptor (Lu and Chow 1999; Park and Poo 2013). This reduction may reflect a loss of trophic support, which could underlie the functional decline observed in prefrontal‐dependent tasks with age (Bruijniks et al. 2020; Oh et al. 2016). PSD‐95 depletion compromises glutamate receptor anchoring and spine structural integrity, and synaptophysin loss implies impaired neurotransmitter release machinery (Sheng and Kim 2011). This pattern aligns with human proteomic studies showing that a reduction in synapse‐associated protein levels correlates with cognitive decline, independent of amyloid pathology (Head et al. 2009). Importantly, AUI rats exhibited these molecular deficits despite preserved behavioral performance, suggesting synaptic protein downregulation may present a latent vulnerability phase preceding overt cognitive impairment.

In the hippocampus, we observed a significant upregulation of BDNF in the absence of any change in synaptophysin or PSD‐95 levels. Importantly, under the experimental conditions used, our western blot results detected a 27–37 kDa band, consistent with the 32‐kDa isoform indicative of proBDNF, while the mature 14‐kDa form was not detected in either brain region. This observation aligns with previous reports that proBDNF is upregulated in the aged rodent hippocampus, although these rats were slightly older (Buhusi et al. 2017; Perovic et al. 2013). While proBDNF can support certain forms of synaptic refinement or developmental plasticity, it has distinct and sometimes opposing functions compared to mature BDNF. Specifically, proBDNF signals through the p75NTR receptor, which can induce pathways associated with synaptic depression, dendritic retraction, or apoptosis, rather than the strengthening and growth typically promoted by mature BDNF. Thus, the observed increase in hippocampal BDNF may reflect age‐associated alterations in BDNF processing or regulation, rather than functional compensation. These findings suggest that molecular responses to aging may differ across brain regions. Together, these region‐specific profiles support the idea that synaptic resilience and plasticity mechanisms may vary between the PFC and hippocampus in aging, with potential consequences for cognitive outcomes.

Next, we investigated age‐related changes in dopaminergic signaling. No significant age‐related changes were observed in the hippocampal expression levels of *Drd1*, *Drd2*, *Comt*, *Net*, and *Dat*. In the PFC, mRNA levels of *Comt* and *Net* were significantly increased in AUI rats compared to young controls, while *Drd1*, *Drd2*, and *Mao‐B* expression remained unchanged. These findings indicate region‐specific, age‐related changes in dopamine clearance mechanisms, with increased expression in catecholamine‐degrading and catecholamine‐transporting enzymes in the PFC. This pattern is consistent with transcriptomic studies showing that gene expression changes in the PFC are highly correlated with age, whereas many hippocampal transcripts remain stable across the lifespan (Dönertaş et al. 2017; Zarrella and Tsurumi 2024). While increased prefrontal dopamine release supports cognitive performance in young animals (McLean et al. 2017), aging is characterized by a shift toward greater dopamine catabolism, as evidenced by the upregulation of *Comt* and *Net*. This transition is not paradoxical but rather reflects an age‐related homeostatic adaptation that ultimately results in reduced dopaminergic tone in the PFC. The resulting hypodopaminergic state is a key contributor to cognitive decline, as supported by evidence that COMT activity influences cortical function across the lifespan (Apud et al. 2006; Tunbridge et al. 2007).

The maintenance of *Drd2* receptor mRNA may contribute to the preservation of recognition memory in AUI rats. Previous studies have shown that hippocampal *Drd2* expression and binding are critical for modulating long‐term potentiation and long‐term depression, both essential for memory (Nyberg et al. 2016; Rocchetti et al. 2015). Human imaging studies further support that *Drd2* binding in the hippocampus and caudate nucleus correlates with recognition memory performance through functional connectivity (Bäckman et al. 2000; Nordin et al. 2021). In contrast, *Drd1* receptor abundance has been linked to the encoding of spatial information in novel environments and aspects of working memory, particularly in novel environments (Lima et al. 2022; Tran et al. 2008; Xing et al. 2010). These findings may explain why deficits in spatial and working memory, as well as cognitive control, often precede declines in recognition memory with aging (Erixon‐Lindroth et al. 2005; Johansson et al. 2023; Liggins 2009; Nyberg 2017).

Given the established link between dopaminergic and inflammatory processes in the aging brain, we next investigated whether age‐related molecular changes in the PFC and hippocampus were associated with alterations in key inflammatory pathways. In the PFC of AUI rats, we found a significant upregulation of *Socs3* and *Nfkb* mRNA, while levels of *Il‐6* and *Cox2* remained unchanged. This pattern is consistent with the literature, as increased Socs3 expression is a well‐recognized marker of enhanced cytokine signaling and negative feedback regulation within the JAK/STAT pathway, often in response to subtle or chronic inflammatory stimuli (Croker et al. 2008). The upregulation of *Nfkb* further points to a shift toward a pro‐inflammatory transcriptional profile, even in the absence of overt increases in classical cytokines such as *Il‐6*. NF‐κB is a transcription factor that, under basal conditions, supports synaptic function and plasticity (Kaltschmidt et al. 2006), but, with increased or chronic activation, can shift to promoting inflammatory responses (Mao et al. 2025). In the hippocampus, we did not detect significant changes in any of these inflammatory markers. This lack of hippocampal *Nfkb* upregulation contrasts with reports of increased NF‐κB signaling in the hippocampus during advanced aging (Porcher et al. 2021; Toliver‐Kinsky et al. 1997). To determine whether age‐related regulation in the hippocampus occurred at the level of transcriptional activity rather than gene expression, we assessed NF‐κB DNA‐binding activity in this region. Despite stable *Nfkb* mRNA expression, we found a significant increase in NF‐κB‐binding activity in the hippocampus of AUI rats compared to young controls. The upregulation of both NF‐κB subunits may represent a compensatory, pro‐survival response to age‐related synaptic stress (Maggirwar et al. 1998). NF‐κB is a key regulator of multiple downstream targets relevant to synaptic health, including PSD‐95 and BDNF. Interestingly, pro‐BDNF, via p75NTR interaction, can induce NF‐κB expression, establishing a complex feedback loop between neurotrophins and inflammatory signaling (Li et al. 2023; Lima Giacobbo et al. 2019; Marini et al. 2004; Nociti and Romozzi 2023). Together, these findings highlight region‐specific differences in inflammatory signaling during ageing, with transcriptional changes evident in the PFC and activity‐level regulation apparent in the hippocampus.

Our data suggest that subtle, region‐specific inflammatory and molecular alterations characterize the aging brain well before overt cognitive decline manifests. The absence of significant changes in *Il‐6* and *Cox2* expression in the PFC, alongside elevated *Socs3*, is consistent with a profile in which classical cytokine or oxidative stress markers are not prominently altered at this stage. Rather than indicating a lack of inflammatory involvement, this pattern may reflect regulatory or compensatory mechanisms operating to maintain inflammatory signaling. Concurrently, increased *Nfkb* expression and elevated NF‐κB‐binding activity point to activation of inflammatory‐related signaling, although these findings are based on gene expression and transcription factor binding and do not allow direct inference of downstream protein activity or functional consequences. Similarly, alterations in BDNF expression suggest changes in synaptic plasticity‐related signaling and in inflammatory homeostasis, but conclusions regarding synaptic function or vulnerability remain necessarily speculative in the absence of protein‐ or receptor‐level analyses. Importantly, unlike classical models of acute neuroinflammation induced by strong immune challenges (e.g., LPS) (Henry et al. 2009; Xie et al. 2003), our findings reflect the subtle, chronic low‐grade inflammation typical of healthy aging. This pattern mirrors the prodromal/high‐risk state observed in neuropsychiatric disorders such as schizophrenia, where early PFC dysfunction precedes clinical symptoms and formal diagnosis (Michalczyk et al. 2023; Misiak et al. 2021; Mongan et al. 2020).

In summary, our study highlights the PFC as a critical early locus of molecular alterations in normal aging, with distinct molecular profiles also observed in the hippocampus, preceding overt cognitive decline. Our findings emphasize the value of targeting the PFC for future investigations into early molecular indicators of neural vulnerability. Although the molecular changes identified here were measured in postmortem brain tissue, the affected pathways, including dopaminergic regulation, synaptic integrity, and inflammatory signaling, are amenable to indirect assessment in living subjects using genetic, peripheral, or imaging‐based approaches. Understanding these early changes may therefore help guide the development of translatable biomarkers and inform strategies for timely intervention aimed at slowing or preventing age‐related cognitive impairment, ultimately improving outcomes for the ageing population.

## Author Contributions

**Junior Bowen**: Formal analysis; investigation; methodology; writing – original draft; writing – review and editing. **Katie Hanna**: Methodology; supervision; writing – review and editing. **Gisela Helfer**: Conceptualization; formal analysis; investigation; methodology; supervision; visualization; writing – original draft; writing – review and editing.

## Funding

The authors have nothing to report.

## Disclosure

This study was first presented at the British Society for Neuroendocrinology meeting in Aberdeen, 2024.

## Conflicts of Interest

The authors declare no conflicts of interest.

## Data Availability

Data are available from the corresponding authors on reasonable request.
