# Correction to “The Role of Dopamine Signaling and Neuroinflammation in Age‐Related Cognitive Dysfunction”

**DOI:** 10.1002/brb3.71519

**Published:** 2026-06-04

Bowen, J., K.Hanna, A. M. J.Young, G.Helfer, and S. L.McLean. 2026. “The Role of Dopamine Signaling and Neuroinflammation in Age‐Related Cognitive Dysfunction.” Brain and Behavior16, no. 4: e71395. https://doi.org/10.1002/brb3.71395

The author contribution section of our paper (https://doi.org/10.1002/brb3.71395) has missed out the contributions of one of the co‐authors A. M. J. Young and S. L. McLean as the senior and corresponding author. The Author Contributions section should read as follows:

## Author Contributions

**Junior Bowen**: formal analysis; investigation; methodology; writing – original draft; writing – review and editing. **Katie Hanna**: methodology; supervision; writing – review and editing. **Andrew Young**: formal analysis; investigation; methodology; writing – review and editing**. Gisela Helfer**: conceptualization; formal analysis; investigation; methodology; supervision; visualization; writing – original draft; writing – review and editing. **Samantha McLean**: Conceptualization; formal analysis; investigation; methodology; supervision; funding acquisition; writing – original draft; writing – review and editing.

We apologize for this error.

